# Supplementary material for: Cilia structure and intraflagellar transport differentially regulate sensory response dynamics within and between C. elegans chemosensory neurons
Source: PLoS Biol. 2024 Nov 26;22(11):e3002892. doi: 10.1371/journal.pbio.3002892 (PMC11593760; doi:10.1371/journal.pbio.3002892)
Supplement: S1 File — (DOCX) [file pbio.3002892.s012.docx]

**Supplementary File 1.** List of strains used in this work.

| **Strain** | **Genotype** | **Source** |
| --- | --- | --- |
| PR802 | *osm-3(p802)* | CGC |
| PY12001 | *osm-3(oy156ts)* | This work |
| MX166 | *che-3(nx159ts)* | (1) |
| VC513 | *grk-2(gk268)* | CGC |
| PY12006 | *osm-6(oy166[gfp_11_])* | This work |
| PY12014 | *kap-1(ok676)*; *osm-3(oy156ts)* | This work |
| PR811 | *osm-6(p811)* | CGC |
| PY12013 | *kap-1(ok676)*; *osm-3(p802)* | This work |
| PY1054 | *oyIs14[sra-6*p::*gfp]* | (2) |
| PY11401 | *osm-6(p811)*; *oyEx[sra-6*p::*gfp]* | (3) |
| PY12029 | *che-3(nx159ts)*; *oyIs14[sra-6*p::*gfp]* | This work |
| PY12030 | *kap-1(ok676)*; *osm-3(oy156ts); oyIs14[sra-6*p::*gfp]* | This work |
| PY12031 | *kap-1(ok676)*; *osm-3(p802)*; *oyIs14[sra-6*p::*gfp]* | This work |
| PY12005 | *kyIs602[sra-6*p::*GCaMP3]* | (4) |
| PY12032 | *kyIs602[sra-6*p::*GCaMP3]; kap-1(ok676)* | This work |
| PY12018 | *kyIs602[sra-6*p::*GCaMP3]; osm-3(p802)* | This work |
| PY12033 | *kyIs602[sra-6*p::*GCaMP3]; daf-10(e1387)* | This work |
| PY12034 | *kyIs602[sra-6*p::*GCaMP3]; osm-6(p811)* | This work |
| PY12019 | *kyIs602[sra-6*p::*GCaMP3]; kap-1(ok676)*; *osm-3(oy156ts)* | This work |
| PY12026 | *kyIs602[sra-6*p::*GCaMP3];che-3(nx159ts)* | This work |
| PY12035 | *osm-6(oy166[gfp_11_])*; *oyEx731[sra-6*p::*gfp_1-10_; sra-6*p::*myr-TagRFP]* | This work |
| PY12007 | *osm-6(oy166[gfp_11_])*; *oyEx682[sra-6*p::*gfp_1-10_]* | This work |
| PY12036 | *kap-1(ok676); osm-3(oy156ts); osm-6(oy166[gfp_11_])*; *oyEx682[sra-6*p::*gfp_1-10_]* | This work |
| PY12037 | *che-3(nx159ts); osm-6(oy166[gfp_11_])*; *oyEx682[sra-6*p::*gfp_1-10_]* | This work |
| PY6345 | *oyEx[sra-6*p::*osm-6*::*gfp]* | (5) |
| PY12023 | *osm-6(oy166[gfp_11_])*; *oyEx681[gpa-4Δ6*p::*gfp_1-10_; gpa-4Δ6*p::*mks-5*::*TagRFP]* | This work |
| PY12024 | *kap-1(ok676); osm-3(oy156ts); osm-6(oy166[gfp_11_]); oyEx681[gpa-4Δ6*p::*gfp_1-10_; gpa-4Δ6*p::*mks-5*::*TagRFP]* | This work |
| PY10210 | *oyIs88[gpa-4Δ6*p::*myr-gfp]* | From A. Maurya |
| PY12038 | *oyIs88[gpa-4Δ6*p::*myr-gfp]; osm-6(p811)* | From A. Maurya |
| PY12039 | *kap-1(ok676)*; *osm-3(p802)*; *oyIs88[gpa-4Δ6*p::*myr-gfp]* | This work |
| PY12028 | *kap-1(ok676)*; *osm-3(oy156ts)*; *oyIs88[gpa-4Δ6*p::*myr-gfp]* | This work |
| PY12027 | *grk-2(gk268)*; *oyIs88[gpa-4Δ6*p::*myr-gfp]* | This work |
| CX14887 | *kyIs598[gpa-6*p::*GCaMP2.2b]* | (6) |
| PY12015 | *kyIs598[gpa-6*p::*GCaMP2.2b]*; *osm-6(p811)* | This work |
| PY12016 | *kyIs598[gpa-6*p::*GCaMP2.2b]*; *kap-1(ok676)*; *osm-3(p802)* | This work |
| PY12022 | *kyIs598[gpa-6*p::*GCaMP2.2b]*; *kap-1(ok676)*; *osm-3(oy156ts)* | This work |
| PY12017 | *kyIs598[gpa-6*p::*GCaMP2.2b]*; *grk-2(gk268)* | This work |
| PY12040 | *kyIs598[gpa-6*p::*GCaMP2.2b]*; *grk-2(gk268)*; *oyEx732[gpa-4Δ6*p::*grk-2*::*tagRFP]* | This work |
| PY12041 | *kyIs598[gpa-6*p::*GCaMP2.2b]*; *bbs-7(jhu590)* | This work |
| PY12042 | *kyIs598[gpa-6*p::*GCaMP2.2b]*; *bbs-7(ok1351)* | This work |
| PY12043 | *ebp-2(oy178[gfp_11_])*; *oyEx681[gpa-4Δ6*p::*gfp_1-10_; gpa-4Δ6*p::*mks-5*::*TagRFP]; oyEx733[gpa-4Δ6*p::*myr-TagRFP]* | This work |
| PY12044 | *ebp-2(oy178[gfp_11_])*; *osm-6(p811)*; *oyEx681[gpa-4Δ6*p::*gfp_1-10_; gpa-4Δ6*p::*mks-5*::*TagRFP]; oyEx733[gpa-4Δ6*p::*myr-TagRFP]* | This work |
| PY12045 | *oyEx734[gpa-4Δ6*p::*kap-1*::*gfp; gpa-4Δ6*p::*myr-TagRFP]* | This work |
| PY12046 | *osm-6(p811)*; *oyEx734[gpa-4Δ6*p::*kap-1*::*gfp; gpa-4Δ6*p::*myr-TagRFP]* | This work |
| PY12047 | *oyEx735[gpa-4Δ6*p::*osm-3*::*gfp; gpa-4Δ6*p::*myr-TagRFP]* | This work |
| PY12048 | *osm-6(p811)*; *oyEx735[gpa-4Δ6*p::*osm-3*::*gfp; gpa-4Δ6*p::*myr-TagRFP]* | This work |
| PY12049 | *oyEx736[gpa-4Δ6*p::*arl-13*::*TagRFP; gpa-4Δ6*p::*myr-gfp]* | This work |
| PY12050 | *osm-6(p811)*; *oyEx736[gpa-4Δ6*p::*arl-13*::*TagRFP; gpa-4Δ6*p::*myr-gfp]* | This work |
| PY12051 | *oyEx737[gpa-4Δ6*p::*odr-3*::*gfp; gpa-4Δ6*p::*myr-TagRFP]* | This work |
| PY12052 | *osm-6(p811)*; *oyEx737[gpa-4Δ6*p::*odr-3*::*gfp; gpa-4Δ6*p::*myr-TagRFP]* | This work |
| PY12053 | *oyEx738[gpa-4Δ6*p::*osm-9*::*gfp; gpa-4Δ6*p::*myr-TagRFP]* | This work |
| PY12054 | *osm-6(p811)*; *oyEx738[gpa-4Δ6*p::*osm-9*::*gfp; gpa-4Δ6*p::*myr-TagRFP]* | This work |
| PY12025 | *srx-64(oy193[gfp_11_])*; *oyEx681[gpa-4Δ6*p::*gfp_1-10_; gpa-4Δ6*p::*mks-5*::*TagRFP]* | This work |
| PY12055 | *srx-64(oy193[gfp_11_])*; *osm-6(p811)*; *oyEx681[gpa-4Δ6*p::*gfp_1-10_; gpa-4Δ6*p::*mks-5*::*TagRFP]* | This work |
| PY12056 | *osm-5(p813)*; *srx-64(oy193[gfp_11_])*; *oyEx681[gpa-4Δ6*p::*gfp_1-10_; gpa-4Δ6*p::*mks-5*::*TagRFP]* | This work |
| PY12057 | *kap-1(ok676)*; *osm-3(p802)*; *srx-64(oy193[gfp_11_])*; *oyEx681[gpa-4Δ6*p::*gfp_1-10_; gpa-4Δ6*p::*mks-5*::*TagRFP]* | This work |
| PY12058 | *bbs-7(jhu590)*; *srx-64(oy193[gfp_11_]); oyEx681[gpa-4Δ6*p::*gfp_1-10_; gpa-4Δ6*p::*mks-5*::*TagRFP]* | This work |
| PY12059 | *bbs-7(ok1351)*; *srx-64(oy193[gfp_11_])*; *oyEx681[gpa-4Δ6*p::*gfp_1-10_; gpa-4Δ6*p::*mks-5*::*TagRFP]* | This work |
| PY12004 | *odr-10(oy158[gfp_11_])*; *oyEx681[gpa-4Δ6*p::*gfp_1-10_; gpa-4Δ6*p::*mks-5*::*TagRFP]* | (7) |
| PY12060 | *odr-10(oy158[gfp_11_])*; *osm-6(p811)*; *oyEx681[gpa-4Δ6*p::*gfp_1-10_; gpa-4Δ6*p::*mks-5*::*TagRFP]* | This work |
| PY12061 | *odr-10(oy158[gfp_11_])*; *kap-1(ok676)*; *osm-3(p802)*; *oyEx681[gpa-4Δ6*p::*gfp_1-10_; gpa-4Δ6*p::*mks-5*::*TagRFP]* | This work |
| PY12062 | *odr-10(oy158[gfp_11_])*; *grk-2(gk268)*; *oyEx681[gpa-4Δ6*p::*gfp_1-10_; gpa-4Δ6*p::*mks-5*::*TagRFP]* | This work |
| PY12063 | *odr-10(oy158[gfp_11_])*; *grk-2(gk268)*; *oyEx681[gpa-4Δ6*p::*gfp_1-10_; gpa-4Δ6*p::*mks-5*::*TagRFP]; oyEx739[gpa-4Δ6*p::*grk-2*::*tagRFP]* | This work |
| PY12064 | *odr-10(oy194[FR])*; *oyEx681[gpa-4Δ6*p::*gfp_1-10_; gpa-4Δ6*p::*mks-5*::*TagRFP]* | This work |
| PY12065 | *odr-10(oy158[gfp_11_])*; *oyEx740[gpa-4Δ6*p::*gfp_1-10_; gpa-4Δ6*p::*myr-TagRFP]* | This work |
| PY12066 | *odr-10(oy158[gfp_11_])*; *kap-1(ok676)*; *osm-3(oy156ts)*; *oyEx740[gpa-4Δ6*p::*gfp_1-10_; gpa-4Δ6*p::*myr-TagRFP]* | This work |
| PY12067 | *odr-10(oy158[gfp_11_])*; *bbs-7(jhu590)*; *oyEx681[gpa-4Δ6*p::*gfp_1-10_; gpa-4Δ6*p::*mks-5*::*tagRFP]* | This work |
| PY12068 | *odr-10(oy158[gfp_11_])*; *bbs-7(ok1351)*; *oyEx681[gpa-4Δ6*p::*gfp_1-10_; gpa-4Δ6*p::*mks-5*::*tagRFP]* | This work |
| PY12069 | *srx-64(oy193[gfp_11_])*; *oyEx740[gpa-4Δ6*p::*gfp_1-10_; gpa-4Δ6*p::*myr-TagRFP]* | This work |
| PY12070 | *kap-1(ok676)*; *osm-3(oy156ts)*; *srx-64(oy193[gfp_11_])*; *oyEx740[gpa-4Δ6*p::*gfp_1-10_; gpa-4Δ6*p::*myr-TagRFP]* | This work |
| PY12071 | *oyIs88[gpa-4Δ6*p::*myr-gfp]*; *oyEx741[gpa-4Δ6*p::*grk-2*::*tagRFP]* | This work |
| PY12072 | *oyIs88[gpa-4Δ6*p::*myr-gfp]*; *osm-6(p811)*; *oyEx741[gpa-4Δ6*p::*grk-2*::*tagRFP]* | This work |
| PY12073 | *kap-1(ok676)*; *osm-3(oy156ts)*; *oyIs88[gpa-4Δ6*p::*myr-gfp]*; *oyEx741[gpa-4Δ6*p::*grk-2*::*tagRFP]* | This work |
| PY12074 | *oyEx742[odr-10*p::*srx-64*::*gfp; gpa-4Δ6*p::*myr-gfp]* | This work |
| PY12075 | *osm-6(p811)*; *oyEx742[odr-10*p::*srx-64*::*gfp; gpa-4Δ6*p::*myr-gfp]* | This work |
| PY12020 | *srx-64(oy195[SL2::gfp_11_])*; *oyEx681[gpa-4Δ6*p::*gfp_1-10_; gpa-4Δ6*p::*myr-TagRFP]* | This work |
| PY12021 | *osm-5(p813)*; *srx-64(oy195[SL2::gfp_11_])*; *oyEx681[gpa-4Δ6*p::*gfp_1-10_; gpa-4Δ6*p::*myr-TagRFP]* | This work |
| PY12080 | *kyIs598[gpa-6*p::*GCaMP2.2b]*; *osm-6(p811)*; *oyEx744[odr-10*p::*srx-64]* | This work |
| PY12076 | *odr-10(oy158[gfp_11_])*; *oyEx743[F16F9.3*p::*mCherry; gpa-4Δ6*p:: *gfp_1-10_]* | This work |
| PY12077 | *odr-10(oy158[gfp_11_])*; *kap-1(ok676)*; *osm-3(oy156ts)*; *oyEx743[F16F9.3*p::*mCherry; gpa-4Δ6*p:: *gfp_1-10_]* | This work |
| PY12078 | *srx-64(oy193[gfp_11_])*; *oyEx743[F16F9.3*p::*mCherry; gpa-4Δ6*p:: *gfp_1-10_]* | This work |
| PY12079 | *kap-1(ok676)*; *osm-3(oy156ts)*; *srx-64(oy193[gfp_11_])*; *oyEx743[F16F9.3*p::*mCherry; gpa-4Δ6*p:: *gfp_1-10_]* | This work |
| PY12097 | *grk-2(gk268) bbs-7(jhu590); kyIs598[gpa-6*p::*GCaMP2.2b]* | This work |

**REFERENCES**

1. Jensen VL, Lambacher NJ, Li C, Mohan S, Williams CL, Inglis PN, et al. Role for intraflagellar transport in building a functional transition zone. EMBO Rep. 2018;19: e45862.

2. Troemel ER, Chou JH, Dwyer ND, Colbert HA, Bargmann CI. Divergent seven transmembrane receptors are candidate chemosensory receptors in *C. elegans*. Cell. 1995;83: 207-218.

3. Cornils A, Maurya AK, Tereshko L, Kennedy J, Brear AG, Prahlad V, et al. Structural and functional recovery of sensory cilia in *C. elegans* IFT mutants upon aging. PLoS Genet. 2016;12: e1006325.

4. Khan M, Hartmann AH, O’Donnell MP, Piccione M, Pandey A, Chao P-H, et al. Context-dependent reversal of odorant preference is driven by inversion of the response in a single sensory neuron type. PLoS Biol. 2022;20: e3001677.

5. Mukhopadhyay S, Lu Y, Qin H, Lanjuin A, Shaham S, Sengupta P. Distinct IFT mechanisms contribute to the generation of ciliary structural diversity in *C. elegans* EMBO J. 2007;26: 2966-2980.

6. Larsch J, Flavell SW, Liu Q, Gordus A, Albrecht DR, Bargmann CI. A circuit for gradient climbing in *C. elegans* chemotaxis. Cell Rep. 2015;12: 1748-1760.

7. Kyani-Rogers T, Philbrook A, McLachlan IG, Flavell SW, O'Donnell MP, Sengupta P. Developmental history modulates adult olfactory behavioral preferences via regulation of chemoreceptor expression in *Caenorhabditis elegans*. Genetics. 2022;222: iyac143.
