# Supplementary material for: Cilia structure and intraflagellar transport differentially regulate sensory response dynamics within and between C. elegans chemosensory neurons
Source: PLoS Biol. 2024 Nov 26;22(11):e3002892. doi: 10.1371/journal.pbio.3002892 (PMC11593760; doi:10.1371/journal.pbio.3002892)
Supplement: S2 File — (DOCX) [file pbio.3002892.s013.docx]

**Supplementary File 2.** List of plasmids used in this work.

| **Plasmid** | **Description** | **Source** |
| --- | --- | --- |
| PSAB1340 | 1kb_genomic*_osm-6_gfp_11__*1kb*_*genomic*_CRISPR* | This work |
| PSAB1341 | 1kb_genomic*_srx-64_gfp_11__1kb_*genomic*_CRISPR* | This work |
| PSAB1349 | 1kb_genomic*_srx-64_SL2*::*gfp_11__*1kb_genomic*_CRISPR* | This work |
| PSAB1280 | *gpa4Δ6*p::*gfp_1-10_* | (1) |
| PSAB1342 | *sra-6*p::*gfp_1-10_* | This work |
| PSAB1343 | *sra-6*p::*myr-TagRFP* | This work |
| PSAB1133 | *gpa4Δ6*p::*myr-TagRFP* | (2) |
| PSAB1120 | *gpa4Δ6*p::*myr-GFP* | (2) |
| PSAB1345 | *gpa4Δ6*p::*grk-2*::*TagRFP* | This work |
| PSAB1144 | *gpa4Δ6*p::*mks-5*::*TagRFP* | (2) |
| PSAB1346 | *gpa4Δ6*p::*osm-9*::*gfp* | This work |
| PSAB1347 | *gpa4Δ6*p::*odr-3*::*gfp* | Ashish Maurya |
| PSAB1126 | *gpa4Δ6*p::*arl-13*::*TagRFP* | (2) |
| PSAB1136 | *gpa4Δ6*p::*kap-1*::*gfp* | (2) |
| PSAB1137 | *gpa4Δ6*p::*osm-3*::*gfp* | (2) |
| PSAB1348 | *odr-10*p::*srx-64*::*gfp* | This work |
| PSAB1355 | *odr-10*p::*srx-64* | This work |
| PSAB1014 | *F16F9.3*p::*mCherry* | (3) |

**REFERENCES**

1. Kyani-Rogers T, Philbrook A, McLachlan IG, Flavell SW, O'Donnell MP, Sengupta P. Developmental history modulates adult olfactory behavioral preferences via regulation of chemoreceptor expression in *Caenorhabditis elegans*. Genetics. 2022;222: iyac143.

2. Maurya AK, Rogers T, Sengupta P. A CCRK and a MAK kinase modulate cilia branching and length via regulation of axonemal microtubule dynamics in *Caenorhabditis elegans*. Curr Biol. 2019;22: 1286-1300.

3. Nechipurenko IV, Olivier-Mason A, Kazatskaya A, Kennedy J, McLachlan IG, Heiman MG, et al. A conserved role for Girdin in basal body positioning and ciliogenesis. Dev Cell. 2016;38: 493-506.
