# Supplementary material for: Cilia structure and intraflagellar transport differentially regulate sensory response dynamics within and between C. elegans chemosensory neurons
Source: PLoS Biol. 2024 Nov 26;22(11):e3002892. doi: 10.1371/journal.pbio.3002892 (PMC11593760; doi:10.1371/journal.pbio.3002892)
Supplement: S1 Table — (DOCX) [file pbio.3002892.s014.docx]

**S1 Table.** Quantification of OSM-6::GFP or OSM-6::split-GFP anterograde movement in ASH cilia.

| **Strain: Genotype** | **Condition^3^** | **Average velocity (μm/sec ± SEM)** | **Average number of IFT events per 30s (± SEM)** | **Number of animals imaged** |
| --- | --- | --- | --- | --- |
| PSAB6345: Ex[*sra-6*p::*osm-6::gfp*] | 20°C | 0.79 ± 0.01 | 15.5 ± 1.36 | 18 |
| PSAB12007: *osm-6(oy166)^1^; oyEx682^2^* | 20°C | 0.81 ± 0.01 | 15.7 ± 0.93 | 24 |
|  | | | | |
| PSAB12007: *osm-6(oy166)^1^; oyEx682^2^* | 20°C | 0.95 ± 0.01 | 23.1 ± 0.56 | 20 |
| PSAB12007: *osm-6(oy166)^1^; oyEx682^2^* | 1 hr 27°C | 0.89 ± 0.01 | 20.5 ± 0.75 | 21 |
| PSAB12007: *osm-6(oy166)^1^; oyEx682^2^* | 1 hr 30°C | 1.01 ± 0.01 | 23.1 ± 0.72 | 22 |
| PSAB12036: *kap-1(ok676); osm-3(oy156ts); osm-6(oy166)^1^; oyEx682^2^* | 20°C | 0.78 ± 0.01 | 19.1 ± 0.58 | 24 |
| PSAB12036: *kap-1(ok676); osm-3(oy156ts); osm-6(oy166)^1^; oyEx682^2^* | 1 hr 27°C | 0.80 ± 0.01 | 15.4 ± 0.93 | 24 |
| PSAB12036: *kap-1(ok676); osm-3(oy156ts); osm-6(oy166)^1^; oyEx682^2^* | 1 hr 30°C | 0.70 ± 0.02 | 3.86 ± 0.81 | 21 |
|  | | | | |
| PSAB12007: *osm-6(oy166)^1^; oyEx682^2^* | 15°C | 0.92 ± 0.01 | 20.6 ± 0.77 | 20 |
| PSAB12007: *osm-6(oy166)^1^; oyEx682^2^* | 3 hr 25°C | 0.86 ± 0.01 | 20.4 ± 0.76 | 22 |
| PSAB12037: *che-3(nx159ts); osm-6(oy166)^1^; oyEx682^2^* | 15°C | 0.84 ± 0.01 | 17.6 ± 0.80 | 20 |
| PSAB12037: *che-3(nx159ts); osm-6(oy166)^1^; oyEx682^2^* | 3 hr 25°C | 0.84 ± 0.01 | 9.55 ± 1.30 | 20 |

^1^*osm-6(oy166): osm-6*::*gfp_11_*

^2^*oyEx682: Ex[sra-6*p::*gfp_1-10_]*

^3^*kap-1; osm-3(ts)* and associated control animals were grown at 20°C prior to the indicated temperature upshifts. *che-3(ts)* and associated control animals were grown at 15°C prior to the indicated temperature upshifts.
