# Supplementary material for: Cilia structure and intraflagellar transport differentially regulate sensory response dynamics within and between C. elegans chemosensory neurons
Source: PLoS Biol. 2024 Nov 26;22(11):e3002892. doi: 10.1371/journal.pbio.3002892 (PMC11593760; doi:10.1371/journal.pbio.3002892)
Supplement: S2 Table — (DOCX) [file pbio.3002892.s015.docx]

**S2 Table.** Quantification of OSM-6::split-GFP anterograde movement in the AWA cilia stalk.

| **Strain: Genotype** | **Condition^3^** | **Average velocity (μm/sec ± SEM)** | **Average number of IFT events per 30s (± SEM)** | **Number of animals imaged** |
| --- | --- | --- | --- | --- |
| PSAB12023: *osm-6(oy166)^1^; oyEx681^2^* | 20°C | 0.60 ± 0.01 | 10.2 ± 1.58 | 22 |
| PSAB12023: *osm-6(oy166)^1^; oyEx681^2^* | 1.5 hr 30°C | 0.64 ± 0.01 | 11.7 ± 1.71 | 18 |
| PSAB12024: *kap-1(ok676); osm-3(oy156ts); osm-6(oy166)^1^; oyEx681^2^* | 20°C | 0.79 ± 0.02 | 5.24 ± 1.35 | 21 |
| PSAB12024: *kap-1(ok676); osm-3(oy156ts); osm-6(oy166)^1^; oyEx681^2^* | 1.5 hr 30°C | 0.60 ± 0.05 | 0.29 ± 0.29 | 21 |

^1^*osm-6(oy166): osm-6*::*gfp_11_*

^2^*oyEx681: Ex[gpa-4Δ6*p::*gfp_1-10_]*

^3^Animals were grown at 20°C prior to the indicated temperature upshifts.
