# Supplementary material for: Cilia structure and intraflagellar transport differentially regulate sensory response dynamics within and between C. elegans chemosensory neurons
Source: PLoS Biol. 2024 Nov 26;22(11):e3002892. doi: 10.1371/journal.pbio.3002892 (PMC11593760; doi:10.1371/journal.pbio.3002892)
Supplement: Attachment — Submitted filename: Philbrook_Reviewer_Comments.docx [file pbio.3002892.s016.docx]

**Response to Reviewers**

**Philbrook et al**

We thank both reviewers for their comments on the manuscript and the Academic Editor for their input. Experimental and textual revisions made in response to their comments are described below.

Reviewer 1

*1. The study claims to have uncovered surprising complexity in the diverse contribution of IFT and cilia morphology to the regulation of responses across individual chemosensory neurons to diverse chemical cues. However, the authors only examine two types of neurons: the ASH nociceptive neuron and the AWA olfactory neuron. Therefore, it is difficult to determine whether the observed differences reflect true mechanistic heterogeneity across various neuronal types or are specific to the differences between ASH and AWA neurons. In addition, the "diverse chemical cues" are limited to two compounds for each neuron, each tested at a single concentration. Although the preliminary data appear promising, it remains uncertain whether the differences in response phenotypes are due to specific chemical structures or differences in chemical concentration.*

*a) To address these concerns, it is essential to conduct dose-response experiments (for all compounds shown in Fig 1B, Figs 2B and 2C).*

Higher concentrations of many alcohols and other odorants that drive avoidance via ASH are too toxic to use at the volumes required for calcium imaging experiments, whereas lower concentrations fail to elicit responses in this neuron. Similarly, low concentrations of many odorants fail to elicit robust responses in AWA (with the exception of diacetyl – please see below), whereas higher concentrations also evoke responses in additional sensory neurons (eg. see (Larsch et al., 2013; Lin et al., 2023), making it difficult to determine whether the responses observed at these high concentrations are cell autonomous (eg. see (Guo et al., 2015; Leinwand and Chalasani, 2013). Since we are specifically interested in responses elicited via cilia, it is critical that we examine responses only to those odorant concentrations previously established by us and others to elicit responses cell-autonomously. We had, therefore, specifically selected concentrations of each chemical that have been shown to elicit strong cell-autonomous responses in ASH or AWA (from the literature and/or our work). However, we appreciate the reviewer’s point and have now examined responses to additional concentrations of multiple chemicals per neuron *within the concentration ranges known to elicit cell-autonomous responses in each neuron type*.

**1. ASH**:

Aqueous chemicals

*1.1) Glycerol:* We had previously reported responses of WT and *osm-6* mutants to 1M glycerol. We have now also examined responses to 0.5M and 2M glycerol (new S1A Fig). Responses of WT and *osm-6* mutants are similar across glycerol concentrations. Thus, while ASH neurons respond robustly to 1M and higher glycerol concentrations in WT animals, these neurons fail to respond in *osm-6* mutants.

*1.2) Quinine:* We have now examined ASH responses to different concentrations of the aversive aqueous chemical quinine (Hilliard et al., 2005). WT but not *osm-6* mutants responded to quinine at both examined concentrations (new S1B Fig).

Volatile chemicals

*1.3) IAA*: We could not perform dose response experiments with IAA since ASH only exhibits responses to 10^-2^ IAA (the concentration used in the manuscript) and not to lower concentrations (Yoshida et al., 2012); higher concentrations are too toxic to be used safely in the large volumes necessary for calcium imaging experiments.

*1.4) 1-heptanol:* We instead examined responses to 10^-3^ and 10^-4^ 1-heptanol in WT and *osm-6* animals (Khan et al., 2022; Lin et al., 2023). ASH maintained a weak response to 10^-3^ concentration of 1-heptanol in *osm-6* mutants similar to their IAA responses, but did not respond to lower concentrations of this chemical (new S1D Fig).

These results further confirm that ASH neurons fail to respond to different concentrations of aqueous chemicals but retain weaker responses to higher concentrations of volatile chemicals in IFT mutants.

**2. AWA**

*2.1) Diacetyl:* Previous work has already reported that *osm-6* mutants retain the ability to respond robustly to a wide range of diacetyl concentrations (Larsch et al., 2015); we now refer to and describe these results in the text.

*2.2) Pyrazine*: We reported responses to 10^-6^ pyrazine in the manuscript. We have now also examined responses to 10^-5^ and 10^-7^ dilutions of pyrazine. Unlike the robust responses of *osm-6* mutants to multiple diacetyl concentrations, responses of *osm-6* mutants were abolished to 10^-7^ pyrazine and significantly reduced to 10^-5^ pyrazine (new Fig 2C, new S3C Fig).

*2.3) 2-heptanone:* We have now also examined responses to different concentrations of 2-heptanone (Lin et al., 2023). Similar to diacetyl responses, *osm-6* mutants retained robust responses to examined concentrations of 2-heptanone and exhibited desensitization defects at both concentrations (new S3B Fig).

*2.4) 2-methylpyrazine:* We now also report responses to different concentrations of 2-methylpyrazine (Larsch et al., 2013). Similar to pyrazine responses, responses of *osm-6* mutants to different concentrations of 2-methylpyrazine were significantly reduced (new S3D Fig).

These results further confirm that AWA retains the ability to robustly respond to a range of concentrations of a subset of chemicals such as diacetyl and 2-heptanone but exhibits desensitization defects to both in IFT mutants. In contrast, AWA exhibits defects in their responses to different concentrations of pyrazine and the related 2-methylpyrazine in IFT mutants.

*b) Additionally, the authors need to increase the number of chemicals tested per receptor type…*

We assume the reviewer meant per neuron type since the ODR-10 and SRX-64 receptors are known to mediate responses primarily to only specific concentrations of diacetyl and pyrazine, respectively.

**ASH**: As described in response to 1a above, we have now examined ASH responses to a total of four chemicals at different concentrations in both WT and *osm-6* mutants. These include glycerol and quinine (aqueous), and IAA and 1-heptanol (volatile). Please see new S1 Fig.

**AWA**: As also described in 1a above, we have now also examined AWA responses to a total of four chemicals at different concentrations (including previously reported diacetyl data -(Larsch et al., 2015)) in both WT and *osm-6* mutants. These include diacetyl, 2-heptanone, pyrazine, and 2-methylpyrazine. Please see new Fig 2C and new S3 Fig.

c) *Therefore, it is difficult to determine whether the observed differences reflect true mechanistic heterogeneity across various neuronal types or are specific to the differences between ASH and AWA neurons……examine at least one additional type of nociceptive neuron and one additional* *type of olfactory neuron in their initial functional characterization (as in Figs 1 and 2).*

We make no claim about generalizing our findings across different neuron types, but very specifically restrict our conclusions to only ASH and AWA. We would like to respectfully state that expanding this work to additional neuron types is beyond the scope of this manuscript.

Individual sensory neuron types in *C. elegans* respond to distinct sets of cues using distinct signaling pathways (Ferkey et al., 2021). Moreover, each olfactory neuron type in the amphid sense organs of the head contain structurally unique cilia built via distinct mechanisms (Doroquez et al., 2014; Evans et al., 2006; Mukhopadhyay et al., 2007; Perkins et al., 1986; Ward et al., 1975). Specifically, the other major nociceptive neuron type in the amphid is the ADL neuron pair; this neuron pair contains two cilia instead of the single cilium in ASH (Doroquez et al., 2014; Perkins et al., 1986; Ward et al., 1975). The two other main olfactory neuron types are AWB and AWC; each have completely different cilia built via distinct IFT mechanisms (Doroquez et al., 2014; Evans et al., 2006; Mukhopadhyay et al., 2007; Perkins et al., 1986; Ward et al., 1975). In addition, AWB and AWC respond in a complex manner to different odorant cues (showing an ‘OFF’ response or both ‘ON/OFF’ responses to different chemicals instead of the ‘ON’ response exhibited by AWA), and AWC in addition exhibits left/right asymmetry, and also responds to thermal cues (Ferkey et al., 2021). Thus, simply examining chemical responses in these neurons in IFT mutants (Figs 1 and 2) will not allow us to draw any meaningful conclusions or further generalize our findings across neuron types. We would need to perform detailed analyses and compare their responses on a neuron-by-neuron basis with conditional inhibition of IFT as we have done here for ASH and AWA.

We have gone through the manuscript and carefully reworded the text to emphasize that our conclusions apply specifically to ASH and AWA and that we are not generalizing across neuron types. We plan to perform similar detailed mechanistic experiments on other neurons with unique cilia structures in the future. To our knowledge, this work represents the first dissection of the contributions of IFT and cilia organization/morphology to chemical responses in a defined chemosensory neuron type in any organism, and we hope that the reviewer agrees that similar neuron-by-neuron analyses will be important subjects for future work.

*2. Fig 1B: The IAA response kinetics also appear to be affected in osm-6, showing a faster decay. Please provide a proper kinetics analysis across various IAA concentrations.*

We have now calculated a t_1/2_ for the desensitization kinetics to IAA in *osm-6* mutants and do find that desensitization kinetics are more rapid (new S1C Fig). As described above, we are unable to examine responses to higher or lower IAA concentrations in ASH. Response amplitudes to 1-heptanol were too low to accurately calculate t_1/2_ (new S1D Fig).

*3. Fig 1B: It appeared that IAA was presented before glycerol. Please tested the reverse sequence to determine whether the order of stimulus presentation affects the phenotype.*

Imaging experiments to different chemicals are not performed sequentially in the same animals. Responses in each animal are only examined once to a single chemical.

*4. Fig 1C: Please perform a proper correlation analysis between the cilium length and response amplitude to support the claim made in Page 6 ("we correlated cilium length with glycerol-evoked Ca2+ dynamics….").*

Since cilia length measurements and calcium responses are not performed in the same animal (challenging due to technical reasons), we cannot provide a direct correlation analysis between cilium length and the response amplitude. We have now edited the text to reflect this.

*5. Fig 1H: Please also test IAA and other chemicals.*

Since Fig 1H shows ASH cilium length in different conditions, we are unsure what the reviewer is referring to?

*6. If the responses of IAA (ASH) to glycerol and IAA are indeed differentially regulated by cilia morphology, what could be the underlying molecular mechanism? It is necessary to test additional aqueous and volatile odorants before any generalization can be made.*

As noted above in response to point 1 above, we have now tested responses in ASH to quinine (aqueous) and 1-heptanol (volatile) in addition to the previously reported glycerol and IAA. Responses to aqueous chemicals (glycerol, quinine) are lost but responses to volatile odorants (IAA, 1-hexanol) are maintained albeit reduced in *osm-6* mutants (new S1 Fig). As described in the Discussion, we speculate that truncation of the ASH cilium in IFT mutants prevents access to signaling molecules by aqueous chemicals, whereas volatile cues may diffuse in through the glial channel to continue to elicit partial responses.

*7. Fig 2B: Is the adaptation phenotype observed only with 30-sec stimulation? How about 10 sec? Fig 2C: Why did the authors change the stimulus duration from 30 sec to 10 sec? Please also provide a kinetics analysis here.*

The desensitization showed in Fig 2B is also observed with a 10s diacetyl stimulus. This is now shown in new S3A Fig. However, since we could not readily fit the data to a t_1/2_ in these conditions, we instead used a 30s stimulus to obtain a more accurate t_1/2_ measurement. Similarly, for the pyrazine response with misexpression of *srx-64* under the *odr-10* promoter, we could not calculate a reliable t_1/2_ measurement to a 10s stimulus, but a t_1/2_ could be calculated for a 30s stimulus as shown in Fig 2G. Note that 10s and 30s stimuli were also used interchangeably by Larsch et al (Larsch et al., 2015). Also see response to point 9 below.

*8. Fig 3A: change the 1.5 hr shift/plus sign to 30⁰C.*

Changed.

*9. Fig 3C: Why did the authors change the stimulus duration from 30 sec to 10 sec? Please also analyze the response kinetics across concentrations.*

As noted above, we observe responses and desensitization to both 10s and 30s stimuli. We used 30s stimuli to obtain t_1/2_ measurements only in Fig 2 since we are unable to obtain t_1/2_ accurate measurements with 10s stimuli under these conditions. Since the AWA cilia are severely truncated in IFT *lof* mutants (Fig 2), it is possible that this difference in cilia morphology contributes in part to these measurement differences. The pulse time is now specifically mentioned in each Figure Legend.

Desensitization kinetics to different diacetyl concentrations have already been described previously (Larsch et al., 2015). As shown in this paper, effective desensitization to diacetyl is observed only in response to a limited concentration range that corresponds to the concentration range at which AWA is required to mediate chemotaxis (Larsch et al., 2015). This is also the case for diacetyl adaptation. We thus selected the concentration (corresponding to 1.15 μM in the Larsch et al paper) that exhibits the most robust and reliable response and desensitization kinetics in our conditions to perform these analyses.

*10. Fig 4C & D: The authors elegantly demonstrated the lack of impact of IFT on nociceptive response and behavior in Fig 1J-K. Please conduct behavioral assays using the inducible mutants (5-hr shift) in a similar manner to demonstrate the specific impact of IFT on olfactory behavior.*

Temperature shifts reported in Fig 1J-K are performed at 25ºC or 27ºC. Temperature shifts reported for AWA (Fig 4C-D) are performed at 30ºC. Animals exposed to 30ºC for 5hr no longer move robustly and we are thus, unable assess their behavior (we tried).

*11) Fig 5E, right panels: It is difficult to visualize EVs in the image.*

We have modified the figure to hopefully make these EVs easier to visualize.

*12. Page 26: The authors stated "these observations support the notion that phosphorylated ODR-10 may be removed from AWA cilia via BBSome-mediated trafficking to mediate diacetyl adaptation". This notion can be strengthened with epistasis experiments to test whether Grk2 phosphorylation of ODR-10 is upstream of BBSome-mediated trafficking.*

We have now compared adaptation to diacetyl in wildtype, *grk-2, bbs-7*, and *grk-2 bbs-7* double mutants. We find that the diacetyl adaptation phenotype of *grk-2 bbs-7* double mutants is similar to that of *grk-2* mutants alone further suggesting that GRK-2 functions upstream of BBS-7-mediated trafficking (new S7F Fig).

*13) Methods: Please specify the age of all experimental animals as the manifestation of phenotypes may be age-dependent.*

Only 1 day old young adults are examined in all experiments. This is now explicitly stated in the first Methods paragraph.

Reviewer 2:

*1. The authors infer that "a minimum cilium length may be necessary for glycerol but not isoamyl alcohol responses in ASH. Alternatively, IFT may differentially regulate responses to these chemicals in ASH". Does IFT affect responses to IAA in ASH? Since isoamyl alcohol is a volatile odorant, it may directly penetrate the worm's cuticle and affect the short cilia, dendrite, or even the cell body. Is it possible that glycerol is not a volatile odorant, so it can only affect the cilia of ASH through the opening channel, thus requiring a longer cilium?*

We do indeed speculate that while volatile odorants can diffuse through the amphid channel to activate ciliary receptors even in truncated ASH cilia, aqueous chemicals are only able to access ciliary receptors when the cilia are sufficiently long to be present at the amphid channel opening. This speculation is now further supported by our analyses of additional aqueous and volatile chemical responses in ASH in wildtype and *osm-6* mutants. As shown in new S1 Fig, we find that *osm-6* mutants retain partial responses to different concentrations of 1-heptanol (in addition to IAA), but fail to respond to quinine (and glycerol). This speculation is explicitly addressed in the Discussion.

*2. Previous studies (Duan et al.,Neuron 2020; Cheng et al., Neuron 2024) have reported that ASH adaptation is mediated in part via GABA release from the AMsh glia. Is it possible that mutations disrupting cilia structure affect glycerol- and/or IAA- responses via changes in AMsh glia- ASH GABAergic signalling?*

To address this possibility, we examined glycerol responses in ASH in wildtype and *osm-6* mutants in the presence of added exogenous GABA, or the GABA_A_ receptor antagonist bicuculline as described (Duan et al., 2020){Wang, 2023 #11751}. As shown in new S1E Fig, we observed no changes in glycerol responses in either WT or *osm-6* mutants, indicating that IFT gene mutations do not affect glycerol responses in ASH via modulation of GABAergic signaling.

*3. Page 22: "While neurons with simple rod-like cilia such as ASE respond to salts over only a 4-fold concentration range, AWA detects diacetyl over a 10^5-fold range." It has been suggested (Taniguchi et al., Science Signaling 2014) that for diacetyl reception, ODR-10 in AWA neurons mediates the attractive response to low concentrations, while SRI-14 in ASH neurons mediates the avoidance response to high concentrations. Is the role and mechanism of cilia structure and IFT similar in regulating AWA responses to both low and high concentrations of diacetyl?*

Previous work (Larsch et al., 2015) has shown that desensitization to diacetyl is observed in response to only a limited concentration range in AWA. For instance, while responses to 1.15 μM diacetyl (the concentration used in this paper) desensitize strongly, responses to 115 μM diacetyl exhibit little desensitization. Similarly, the extent of adaptation is markedly decreased at higher diacetyl concentrations (Larsch et al., 2015). Experimental analyses in this paper showed that the diacetyl concentration ranges at which desensitization and adaptation are observed correspond to the concentration ranges at which the AWA neurons are required for chemotaxis (Larsch et al., 2015). Given the weak desensitization and adaptation observed in response to high diacetyl concentrations in wildtype AWA neurons, we cannot reliably assess the effects of IFT mutations on these phenotypes. We now specifically describe our reasoning for selecting the examined odorant concentrations in our assays in the Methods.

*In summary,* in response to the reviewers’ comments, we made revisions to the text and figures and added extensive new experimental data and analyses. New data and analyses are now shown in new Figures 2C, S1A-E, S3A-D, and S7F. We have also revised Figures 3A, S3, S4 and 5E. We thank the reviewers for their comments and interest and hope that they find the revisions satisfactory.

**REFERENCES**

Doroquez, D.B., Berciu, C., Anderson, J.R., Sengupta, P., and Nicastro, D. (2014). A high-resolution morphological and ultrastructural map of anterior sensory cilia and glia in *C. elegans*. eLife *3*, e01948.

Duan, D., Zhang, H., Yue, X., Fan, Y., Xue, Y., Shao, J., Ding, G., Chen, D., Li, S., Cheng, H.*, et al.* (2020). Sensory glia detect repulsive odorants and drive olfactory adaptation. Neuron *108*, 707-721 e708.

Evans, J.E., Snow, J.J., Gunnarson, A.L., Ou, G., Stahlberg, H., McDonald, K.L., and Scholey, J.M. (2006). Functional modulation of IFT kinesins extends the sensory repertoire of ciliated neurons in *Caenorhabditis elegans*. J Cell Biol *172*, 663-669.

Ferkey, D.M., Sengupta, P., and L'Etoile, N.D. (2021). Chemosensory signal transduction in *Caenorhabditis elegans*. Genetics *217*, iyab004.

Guo, M., Wu, T.H., Song, Y.X., Ge, M.H., Su, C.M., Niu, W.P., Li, L.L., Xu, Z.J., Ge, C.L., Al-Mhanawi, M.T.*, et al.* (2015). Reciprocal inhibition between sensory ASH and ASI neurons modulates nociception and avoidance in *Caenorhabditis elegans*. Nat Commun *6*, 5655.

Hilliard, M.A., Apicella, A.J., Kerr, R., Suzuki, H., Bazzicalupo, P., and Schafer, W.R. (2005). In vivo imaging of *C. elegans* ASH neurons: cellular response and adaptation to chemical repellents. EMBO J *24*, 63-72.

Khan, M., Hartmann, A.H., O’Donnell, M.P., Piccione, M., Pandey, A., Chao, P.-H., Dwyer, N.D., Bargmann, C.I., and Sengupta, P. (2022). Context-dependent reversal of odorant preference is driven by inversion of the response in a single sensory neuron type. PLoS Biol *20*, e3001677.

Larsch, J., Flavell, S.W., Liu, Q., Gordus, A., Albrecht, D.R., and Bargmann, C.I. (2015). A circuit for gradient climbing in *C. elegans* chemotaxis. Cell Rep *12*, 1748-1760.

Larsch, J., Ventimiglia, D., Bargmann, C.I., and Albrecht, D.R. (2013). High-throughput imaging of neuronal activity in *Caenorhabditis elegans*. Proc Natl Acad Sci USA *110*, E4266-4273.

Leinwand, S.G., and Chalasani, S.H. (2013). Neuropeptide signaling remodels chemosensory circuit composition in *Caenorhabditis elegans*. Nat Neurosci *16*, 1461-1467.

Lin, A., Qin, S., Casademunt, H., Wu, M., Hung, W., Cain, G., Tan, N.Z., Valenzuela, R., Lesanpezeshki, L., Venkatachalam, V.*, et al.* (2023). Functional imaging and quantification of multineuronal olfactory responses in *C. elegans*. Sci Adv *9*, eade1249.

Mukhopadhyay, S., Lu, Y., Qin, H., Lanjuin, A., Shaham, S., and Sengupta, P. (2007). Distinct IFT mechanisms contribute to the generation of ciliary structural diversity in *C. elegans* EMBO J *26*, 2966-2980.

Perkins, L.A., Hedgecock, E.M., Thomson, J.N., and Culotti, J.G. (1986). Mutant sensory cilia in the nematode *Caenorhabditis elegans*. Dev Biol *117*, 456-487.

Ward, S., Thomson, N., White, J.G., and Brenner, S. (1975). Electron microscopical reconstruction of the anterior sensory anatomy of the nematode *Caenorhabditis elegans.* J. Comp. Neurol. *160*, 313-337.

Yoshida, K., Hirotsu, T., Tagawa, T., Oda, S., Wakabayashi, T., Iino, Y., and Ishihara, T. (2012). Odour concentration-dependent olfactory preference change in *C. elegans*. Nat Commun *3*, 739.
